# Reaffirming and Clarifying the American Society of Clinical Oncology’s Policy Statement on the Critical Role of Phase I Trials in Cancer Research and Treatment

**DOI:** 10.1200/JCO.2016.70.4692

**Published:** 2016-11-28

**Authors:** Jeffrey S. Weber, Laura A. Levit, Peter C. Adamson, Suanna S. Bruinooge, Howard A. Burris, Michael A. Carducci, Adam P. Dicker, Mithat Gönen, Stephen M. Keefe, Michael A. Postow, Michael A. Thompson, David M. Waterhouse, Susan L. Weiner, Lynn M. Schuchter

**Affiliations:** Jeffrey S. Weber, New York University Langone Medical Center, New York, NY; Laura A. Levit, American Society of Clinical Oncology, Alexandria, VA; Peter C. Adamson, The Children's Hospital of Philadelphia, Philadelphia, PA; Suanna S. Bruinooge, American Society of Clinical Oncology, Alexandria, VA; Howard A. Burris, Sarah Cannon Research Institute, Nashville, TN; Michael A. Carducci, Sidney Kimmel Comprehensive Cancer Center, Baltimore, MD; Adam P. Dicker, Jefferson Medical College of Thomas Jefferson University, Philadelphia, PA; Mithat Gönen, Memorial Sloan Kettering Cancer Center, New York, NY; Stephen M. Keefe, University of Pennsylvania, Philadelphia, PA; Michael A. Postow, Memorial Sloan Kettering Cancer Center, New York, NY; Michael A. Thompson, Aurora Health Care, Milwaukee, WI; David M. Waterhouse, Oncology Hematology Care, Cincinnati, OH; Susan L. Weiner, Children's Cause for Cancer Advocacy, Washington, DC; and Lynn M. Schuchter, University of Pennsylvania, Philadelphia, PA

On behalf of ASCO, we thank Dr Jonathan Kimmelman for highlighting a number of important issues in the design and conduct of phase I clinical trials in oncology.^1^ The ethics surrounding cancer phase I trials have been an important topic of discussion throughout the modern history of clinical cancer research and continue to be important in the development of new drugs. We would like to reaffirm and clarify ASCO’s position on phase I cancer clinical trials^2^ and to agree or disagree with some of Dr Kimmelman’s points.

ASCO and Dr Kimmelman agree that phase I trials have therapeutic intent and that such intent is necessary, but not sufficient, to support conduct of such trials in patients with cancer. As noted in ASCO’s 2015 policy statement update on this topic,^2^ these trials must also have the potential to provide clinical benefit. In offering an interventional trial, the physician and patient have the goal of attempting to treat the cancer. The same goal applies when a physician and patient pursue therapeutic options outside a clinical trial.

Dr Kimmelman challenges the underlying assumptions and evidence on which the potential therapeutic benefit of participation in phase I trials rests. His reasoning begins with the tenet that the risk-benefit assessment is always made against the current standard of care. However, the risk-benefit assessment must also take into account the disease setting, which necessarily includes the known risks associated with progressive cancer. The standard of care for patients who participate in phase I trials usually is either to forego anticancer treatment and pursue symptom management or to receive off-label cancer drugs with little proven effectiveness. Trial participation has the advantage over standard care of generating data on patient outcomes, safety, and efficacy for therapies. ASCO supports the registration of all trials in the national trials registry (ClincialTrials.gov) and the publication of research results to ensure these data are available to the public.^3^

In addition, ASCO wishes to again emphasize the evolving landscape of cancer drug development and its commitment to protecting human patients and ensuring the ethical conduct of cancer research. Innovative phase I trial designs can and should limit the risks of patients receiving a dose of a drug that is too low to be effective. The use of biomarker selection strategies can increase the probability of obtaining clinical benefit. Indeed, emerging data show that biomarker-directed therapies are associated with improved response and progression-free survival in the phase I setting,^4^ and it is no longer uncommon to offer patients the option of a phase I trial even before all standard treatment approaches have been used. The recent history of oncology trials, particularly in the field of immunotherapy, suggests that patients have clearly benefited from early-phase trials of drugs such as ipilimumab, nivolumab, pembrolizumab, and atezolizumab, and the trials that led to their approval were in some cases the actual phase I studies that were extended to include a broader patient population.^5^ According to a recent estimate, approximately two thirds of agents tested in phase I trials proceed to the next phase of development.^6^

Although response rate may in some cases be a poor surrogate for overall survival, response rate is typically accepted by the US Food and Drug Administration as an end point reasonably likely to predict clinical benefit and thus provide a basis for accelerated approval. In fact, several of these recent approvals were based on response data that ultimately were translated into improved survival outcomes and prominent long-term tails on the survival curve in later-phase randomized studies.^7^

Patients considering participation in any type of trial need understandable information about the uncertainty for potential benefit and risk^8^ as well as the research objectives of the study. Given the evolving landscape of cancer drug development, ASCO recommends that informed consent documents and patient educational materials rely on peer-reviewed data to present the known and unknown benefits and risks of an investigational early-phase agent. These documents should be written at an 8th grade reading level, because many current forms are long, complex, and highly technical.^9^ ASCO continually advocates that informed consent forms be simplified to optimize comprehensibility and clarity, reduce intimidating language, and place potential benefits and risks in a proper context.^10^ In addition, it is critical that clinicians discuss this information in the informed consent process with the goal of ensuring that potential trial participants and their family members make fully informed decisions about whether to enroll in the trial.
